# Impact of Caveolin-Mediated Endocytosis on the Trafficking of HIV within the Colonic Barrier

**DOI:** 10.1128/jvi.00202-22

**Published:** 2022-03-17

**Authors:** Alex Anwar, Michelle Helou, Jessica Hervol, Alan D. Levine

**Affiliations:** a Department of Molecular Biology and Microbiology, Case Western Reserve University, Cleveland, Ohio, USA; b Department of Pathology, Case Western Reserve University, Cleveland, Ohio, USA; c Department of Pharmacology, Case Western Reserve University, Cleveland, Ohio, USA; d Department of Medicine, Case Western Reserve University, Cleveland, Ohio, USA; e Department of Pediatrics, Case Western Reserve University, Cleveland, Ohio, USA; f Department of Biological Sciences, Case Western Reserve University, Cleveland, Ohio, USA; g CWRU Center for Excellence on the Impact of Substance Use on HIV, Case Western Reserve University, Cleveland, Ohio, USA; Emory University

**Keywords:** HIV, transcytosis, intestinal epithelial cells, lipid rafts, lysosomes, transepithelial resistance, human immunodeficiency virus

## Abstract

In the United States, most new cases of human immunodeficiency virus (HIV) belong to the at-risk group of gay and bisexual men. Developing therapies to reverse viral latency and prevent spread is paramount for the HIV cure agenda. In gay and bisexual men, a major, yet poorly characterized, route of HIV entry is via transport across the colonic epithelial barrier. While colonic tears and paracellular transport contribute to infection, we hypothesize that HIV entry through the colonic mucosa proceeds via a process known as transcytosis, involving (i) virion binding to the apical surface of the colonic epithelium, (ii) viral endocytosis, (iii) transport of virions across the cell, and (iv) HIV release from the basolateral membrane. Using Caco-2 colonic epithelial cells plated as a polarized monolayer in transwells, we characterized the mechanism of HIV transport. After exposing the monolayer to HIV apically, reverse transcription quantitative PCR (RT-qPCR) of the viral genome present in the basolateral chamber revealed that transport is dose dependent, cooperative, and inefficient, with released virus first detectable at 12 h. Inefficiency may be associated with >50% decline in detectable intracellular virus that correlates temporally with increased association of the virion with lysosomal-associated membrane protein 1 (LAMP-1+) endosomes. Microscopy revealed green fluorescent protein (GFP)-labeled HIV within the confines of the epithelial monolayer, with no virus detectable between cells, suggesting that viral transport is transcellular. Treatment of the monolayer with endocytosis inhibitors, cholesterol reducing agents, and small interfering RNA (siRNA) to caveolin showed that viral endocytosis is mediated by caveolin-coated endosomes contained in lipid rafts. These results indicate that HIV transport across the intestinal epithelial barrier via transcytosis is a viable mechanism for viral spread and a potential therapeutic target.

**IMPORTANCE** Despite the success of combination antiretroviral therapy in suppressing HIV replication and the emergence and effectiveness of PrEP-based prevention strategies, in 2018, 37,968 people in the United States received a new HIV diagnosis, accompanied by 15,820 deaths. While the annual number of new diagnoses decreased 7% from 2014 to 2018, 14% of people with HIV did not know they were infected. Gay and bisexual men accounted for 69% of all HIV diagnoses and 83% of diagnoses among males. Due to the scope of the HIV epidemic, determining and understanding precise routes of infection and the mechanisms of viral spread are paramount to ending the epidemic. Since transcellular transport of HIV across an intact colonic epithelial barrier is poorly understood, our overall goal is to characterize the molecular events involved in HIV transcytosis across the intestinal epithelial cell.

## INTRODUCTION

In 2018, roughly 1.7 million people died from human immunodeficiency virus (HIV)-related causes worldwide, accompanied by 770,000 deaths caused by AIDS-related illnesses (1). Educational, sociological, and clinical approaches are viable options for preventing the spread of HIV, which is the major goal of the End the HIV Epidemic (EHE) program (2). The greatest at-risk group for HIV transmission in the United States is gay, bisexual, and transgender men (1). Due to the nature of these men’s sexual contact, the rectal and colonic epithelium is a key site for HIV entry. There are several proposed routes of HIV transmission through the epithelium: colonic tears, intercellular transport, and intracellular transport (3, 4). The relative contribution of colonic tears and intercellular transport was reviewed recently (5), revealing that these modes of HIV transport across the colonic barrier are not as prevalent as once thought.

Transcytosis is the process of transporting macromolecules across a cell using endosomal vesicles (6–8). The best-studied example of transcytosis is transport of IgA across gut epithelial cells from the lamina propria into the lumen (7, 9). IgA binds to the poly immunoglobulin receptor (pIgR), is endocytosed, and is transported and released from the apical surface of the cell. PIgR cleaves itself and releases a dimeric IgA/pIgR complex into the colonic mucus (10). While IgA transcytosis is from basal to apical surfaces, apical to basal transcytosis has also been documented for leptin and Epstein-Barr Virus (EBV) (11, 12). Leptin binds to the apical leptin receptor (both long and short versions of the receptor) and is endocytosed in a clathrin-dependent manner. Transport of the endosome to the Golgi apparatus occurs and leptin is released from the basal membrane attached to a soluble form of the receptor (11). EBV also undergoes transcytosis across oral and gut epithelial cells, capable of both apical to basal and basal to apical transit (12). During apical to basal transcytosis, EBV enters through the epithelial cell’s macropinocytosis mechanism (which does not require a receptor) and eventually collects in cellular endosomes. Despite the parallels with infective, directional apical to basal transport of EBV, HIV transcytosis seems to be more similar mechanistically to the process EBV utilizes for basal to apical transcytosis (13).

While HIV transcytosis is not as well characterized as that of EBV, HIV is reported to undergo transcytosis across epithelial cells, leading to productive infection of T cells (14). Previous studies have proposed that surface receptors, such as galactosylceramide and the heparan sulfate proteoglycan family (15), play a role in binding and uptake of the virions (14–17). Downstream mechanisms of HIV transcytosis that have previously been proposed include the use of the endocytic recycling pathway (13) and neutralizing antibodies against HIV to aid in transport; however, the latter is currently controversial (18). Intact HIV virions can be endocytosed into vaginal epithelial cells via the endocytic recycling pathway, and recycling endosomal inhibitors reduces the rate of viral entry. HIV has been found to associate with late endosomes, specifically becoming sequestered within them over time, potentially leading to viral release after epithelial-lymphocyte interactions (19, 20). It was previously shown that HIV released from the basolateral surface of an intact monolayer is capable of infection, since the envelope remains intact during transport (14). Currently, controversy surrounds whether broadly neutralizing antibodies (IgA and IgG) play a role in viral transport across epithelial cells (21, 22). Theoretically, broadly neutralizing antibodies found in the mucus of the lumen may bind to HIV (23), aiding the virus in transcytosis across the cell with help from Fc receptors on the apical surface of the cells (21). Virally bound IgA can reportedly aid in neutralization of infectious virus post transcytosis (24), leading to viral uptake by T cells, and finally degradation (18) or infection (14), depending on the study.

Endocytosis, the process of materials being brought into the cell, is facilitated by several pathways: a clathrin pathway, a caveolin pathway, and a non-clathrin-caveolin pathway (25). The clathrin pathway of endocytosis is characterized by an invagination coated by the clathrin protein (binding to AP-2), thus stabilizing the invagination as it continues to form. Once the invagination has severed from the plasma membrane and is stable, the clathrin will unbind and be recycled. In a similar fashion, the caveolin-based pathway of endocytosis involves the formation of an invagination from the plasma membrane being coated with caveolin and cavin-1 (26). These proteins stabilize the caveolae as it forms but do not fall off once the vesicle is fully formed. We hypothesize that HIV may be utilizing one or both pathways to enable transport across epithelial cells.

While HIV transcytosis has been shown to occur in both genital and colonic epithelial cells (13, 14), these studies lack detailed investigation of downstream mechanisms. To model the colonic epithelium for studies on HIV transport, the human intestinal epithelial colorectal adenocarcinoma cell line, Caco-2, was grown in a transwell culture, where it spontaneously forms a polarized, intact, semipermeable monolayer (14, 27–33). We found that HIV transcytosis occurs in the apical to basal direction within 12 h of incubation with HIV, as detected by reverse transcription quantitative PCR (RT-qPCR) for the viral genome and microscopy. We identified caveolin-mediated endocytosis as a primary pathway of HIV uptake into the epithelial monolayer using pharmacological inhibitors and small interfering RNA (siRNA) silencing, as well as 3D image visualizations and infectivity assays, to confirm that infectious viral particles were located within the cells of the monolayer.

## RESULTS

### Epithelial monolayer model for HIV transport.

**FIG 1:**
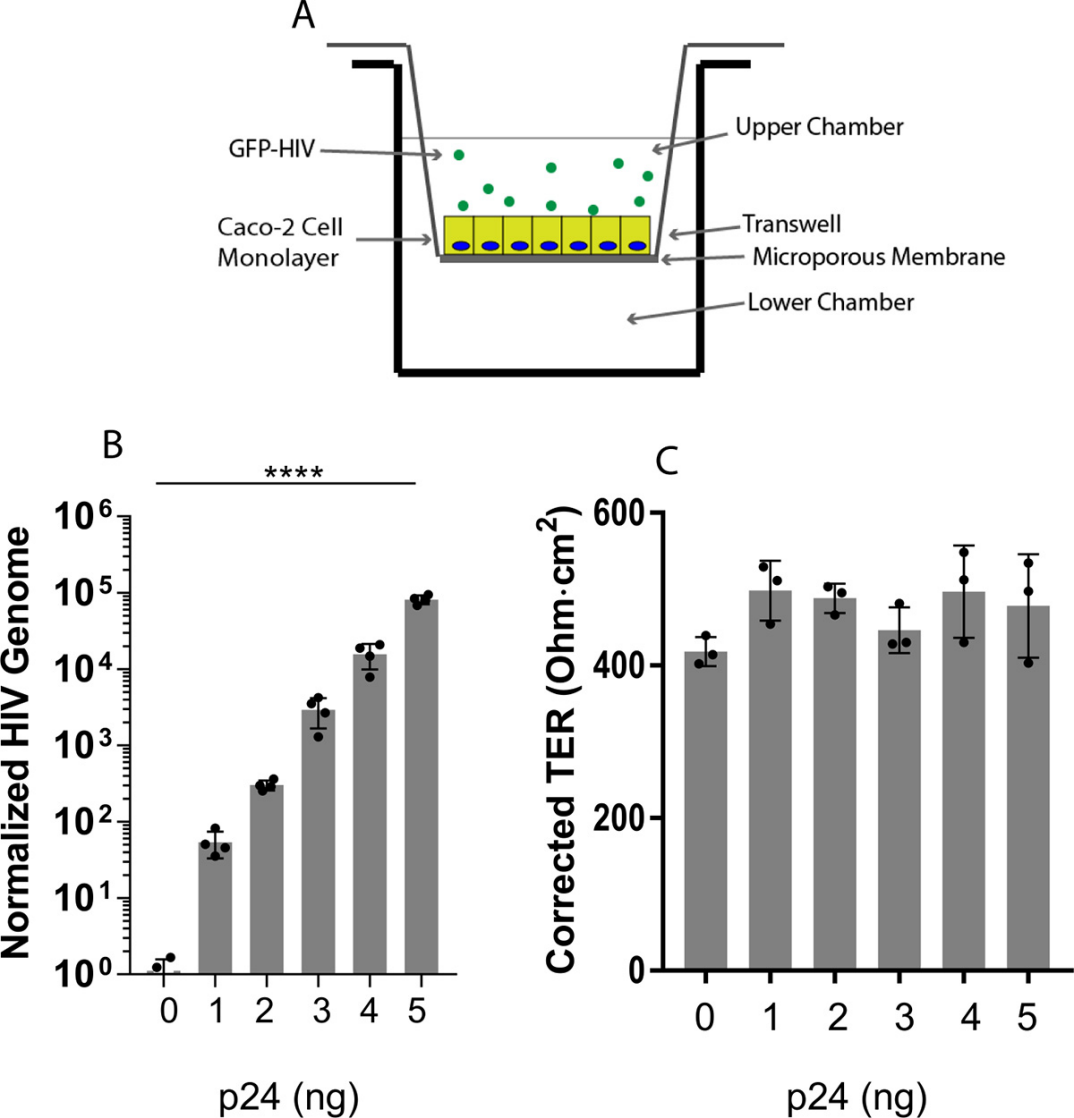
HIV transport across an intestinal epithelial cell monolayer. (A) Caco-2 cells, a human colonic epithelial adenocarcinoma cell line, were plated on the membrane of a transwell chamber, allowed to polarize for 7 to 10 days, and then incubated with GFP-labeled HIV in the upper chamber for 2 h. (B) HIV release into the lower chamber was measured at 24 h by RT-qPCR for the viral genome; ****, *P* ≤ 0.0001 by ANOVA. (C) Transepithelial resistance (TER), recorded at 12 h, is unchanged during the transport of HIV across the monolayer.

To establish a model for HIV transport across a colonic epithelial monolayer, we used polarized Caco-2 epithelial cells grown in a transwell culture plate. The cells were plated on the upper surface of the transwell membrane and allowed to polarize for 7 to 10 days (Fig. 1A). Monolayers were tested for transepithelial resistance (TER) every other day and used when they stabilized at ∼400 to 500 Ω·cm^2^. Cells were stained for occludin, a tight junction protein, and the monolayer was confirmed to be intact when a honeycomb pattern was observed (data not shown). Graded concentrations of GFP-labeled HIV were added to the apical surface for 2 h and then removed, and the cell monolayer was incubated for 24 h. After incubation, the basolateral medium was collected and viral RNA was isolated. A fixed amount of purified cellular RNA was added as an internal control. cDNA was synthesized from the mixture of viral and host RNA by reverse transcription, and the cDNA was probed with primers targeting the reverse transcriptase (RT) gene of HIV. Quantification of the qPCR results, normalized to cellular GAPDH, shows that HIV is released from the colonic epithelium in a dose-dependent manner (Fig. 1B). TER readings taken at each concentration of virus revealed that monolayer integrity was not compromised by the HIV virion (Fig. 1C) and that in the absence of the epithelial cell monolayer, HIV diffused freely across the transwell microporous membrane (Fig. S1).

### HIV passes through the monolayer in a time-dependent manner.

**FIG 2:**
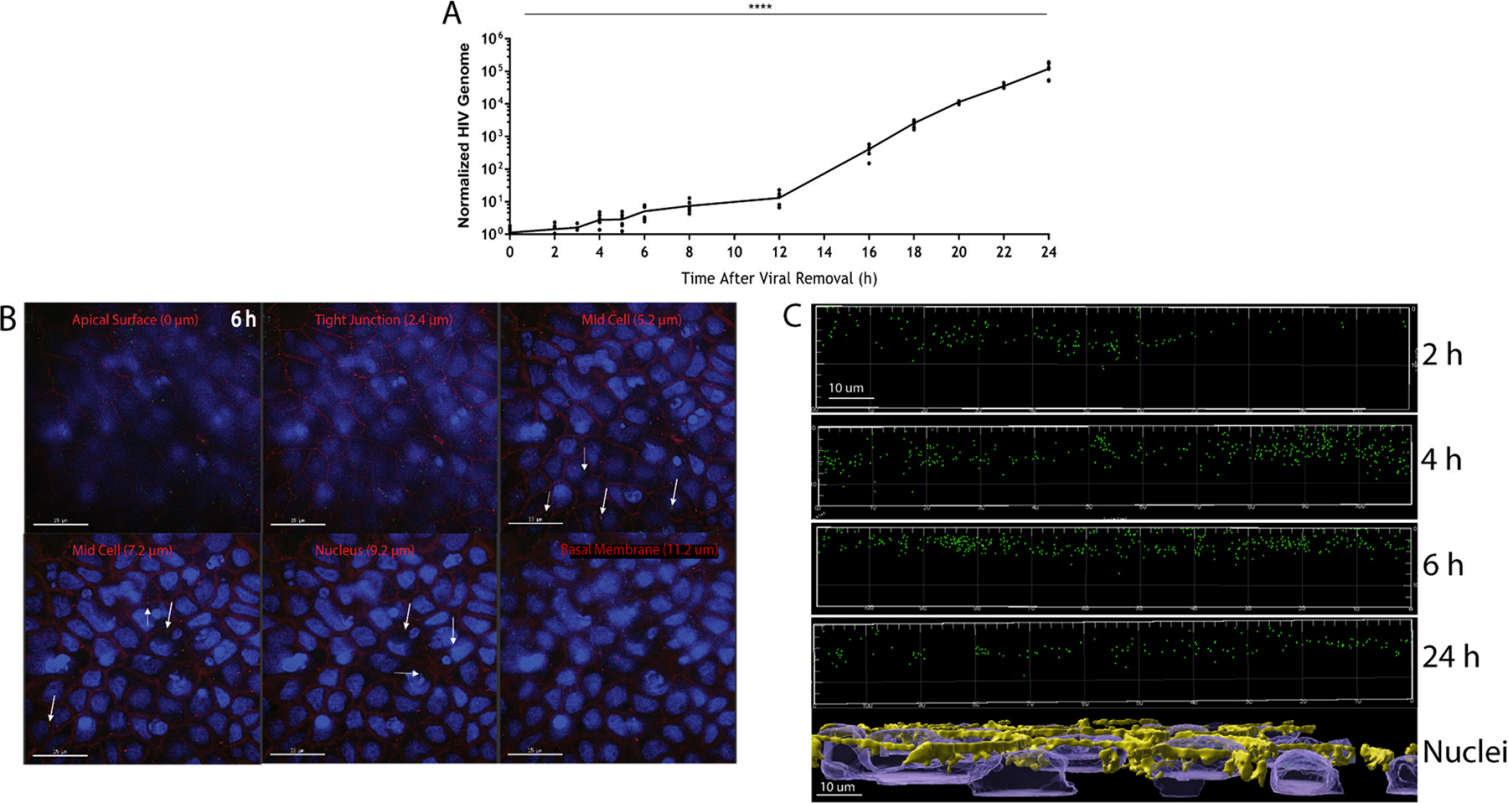
HIV internalization, transport, and release are time dependent. (A) Polarized epithelial monolayers in transwells were incubated with GFP-HIV for 2 h. Viral release into the lower chamber was followed by RT-qPCR over 24 h; ****, *P* ≤ 0.0001 by ANOVA. (B) GFP-HIV particles are found throughout the epithelial cell monolayer at 6 h. Viral particles (white arrows) were imaged at different depths of the columnar epithelium using deconvolution fluorescence microscopy. (Green, GFP-HIV; red, occludin; blue, DAPI). (C) Visualization of the z-stacks at 2, 4, 6, and 24 h imaged along the XZ plane reveals the depth of viral entry into the Caco-2 cell monolayer. “Nuclei” image shows side view of representative monolayer imaged. Yellow, occludin; blue, DAPI.

To follow the kinetics of HIV transport across the monolayer, we exposed the cells to 5 ng of p24 of GFP-HIV for 2 h and then sampled them over a 24-h period, collecting the basolateral media for HIV genome analysis and staining the transwell inserts for microscopy. Significant viral RNA release into the basolateral medium was first detected at 12 h and increased steadily for the next 12 h (Fig. 2A). TER of each transwell was measured over 24 h, confirming that the monolayers remained intact (Fig. S2). Transwell inserts at 2, 4, 6, 12, and 24 h were fixed using 4% paraformaldehyde, and the cells were stained for occludin (a tight junction protein) and DAPI (4′,6-diamidino-2-phenylindole). Using deconvolution fluorescence microscopy, we generated Z-stacks of the monolayers. GFP-labeled viral particles were detected across the entire breadth of the polarized cell within 6 h (Fig. 2B, Movie S1). Time points at 2, 4, and 18 h were also imaged and confirmed the broad distribution of virus within the cell (Fig. S3 to S5). To further evaluate viral progression through the monolayer, 3D visualizations of the Z-stacks were compiled (IMARIS 9.2.2) and XZ plots were prepared. These visualizations revealed that over the 24-h period of transport, the virions appear to collect within the upper half of the monolayer, with few viruses transported deeper (Fig. 2C). An XZ-view of the culture, stained for occludin and nuclei (Fig. 2C), and a top-down view of the culture (Fig. S6) confirm the presence of a single epithelial cell layer within the polarized monolayer.

**FIG 3:**
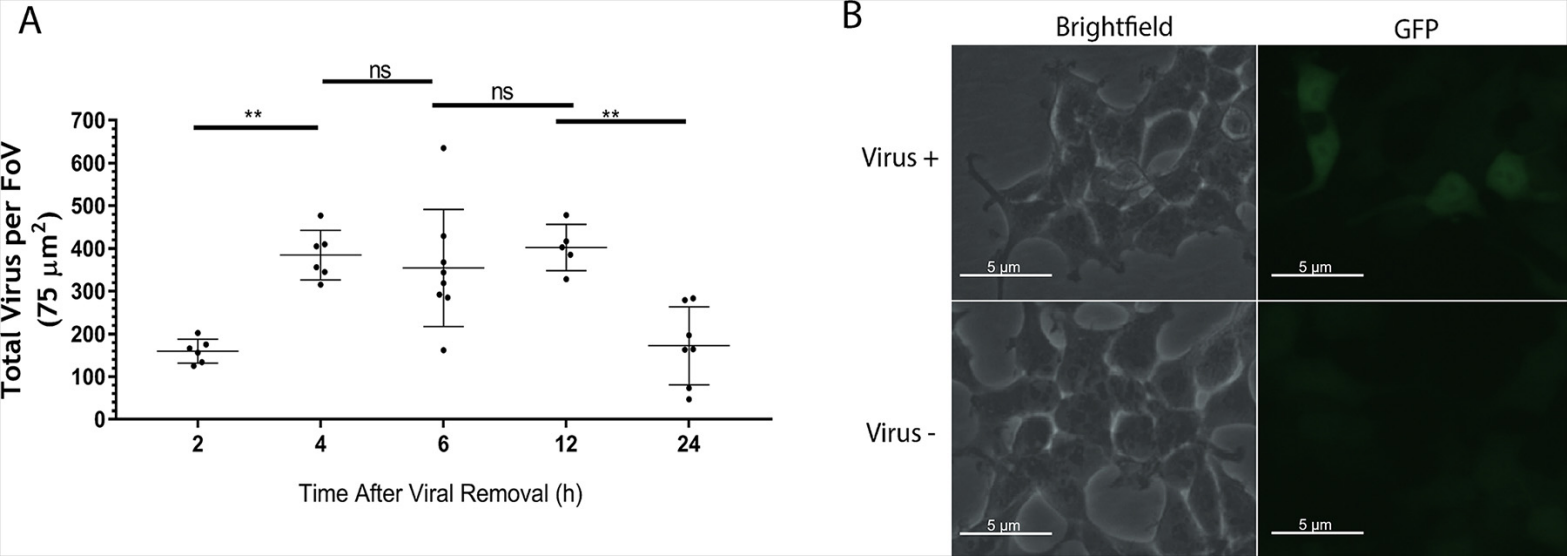
HIV within the monolayer is reduced over 24 h but remains infectious. (A) Individual virions found within the monolayer were counted using IMARIS and quantified over time; **, *P* < 0.01 using ANOVA with Bonferroni correction. (B) Live, intact virus was extracted from the monolayer and incubated with Ghost X4/R5 cells for 72 h. Ghost cells successfully infected are indicated by presence of GFP.

Quantification of virions within the monolayer reveals that intracellular virion concentrations drop precipitously between 12 and 24 h, suggesting that the virions may be degrading (Fig. 3A). Virions were isolated from the monolayer at 12 h, before their potential destruction, and tested for infectivity. Whole cell lysate was cultured with Ghost (3) X4/R5-tropic cells for 72 h. The presence of a GFP signal indicates that intact, infective virions are present within the monolayer (Fig. 3B).

To investigate the energetics of viral transport, we incubated 5 ng of p24 of HIV on polarized Caco-2 monolayers for 2 h at either 37°C or 4°C. Unbound virus was then removed, and fresh medium was added to the apical chamber. The cells were then allowed to incubate for a further 24 h. Basolateral medium was collected, and RT-qPCR analysis was performed as described previously (Fig. S7). Transport of virions incubated at 4°C was reduced by 80% compared to that at 37°C, suggesting that this process is energy dependent.

### 3D visualization of monolayers and bald virus reveals predominantly intracellular transport.

**FIG 4:**
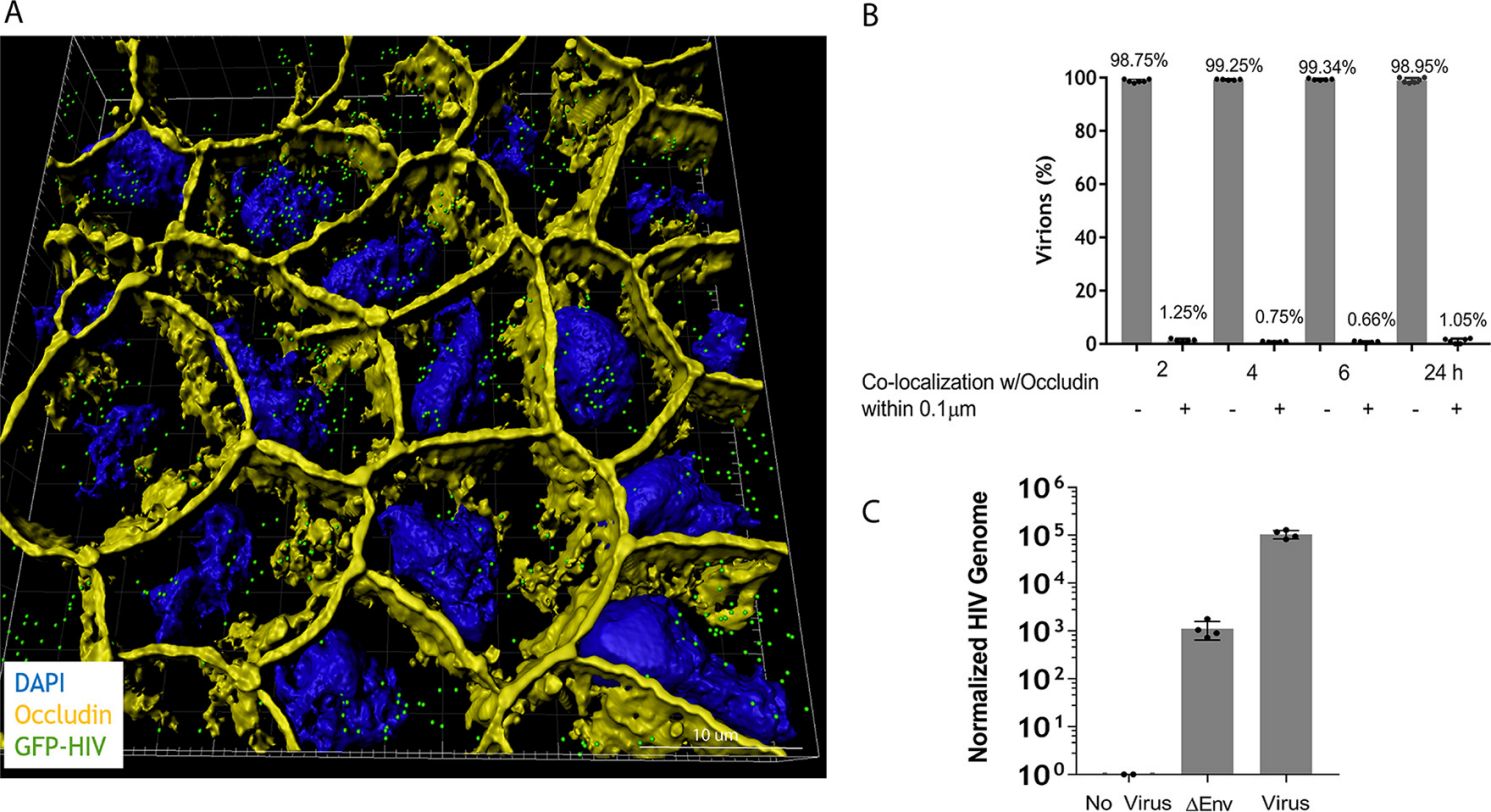
3D visualization of polarized epithelial monolayers and bald virus reveals that HIV is found within the confines of the cell. (A) 3D visualizations of GFP-HIV within a polarized Caco-2 monolayer at 6 h using a z-stack generated with IMARIS (green, GFP-HIV; yellow, occludin; blue, DAPI). (B) Colocalization quantification panels representative of panel A (*n* = 6). (C) Five nanograms of p24 of Δenvelope GFP-HIV was incubated in the upper chamber of an intact epithelial cell monolayer for 2 h. HIV release into the lower chamber was measured at 24 h by RT-qPCR.

To study the localization of virus within the monolayer, we compiled the deconvolution microscopic Z-stacks taken at 6 h by the 3D visualization software IMARIS (Fig. 4A). This revealed that the vast majority of virions were located within the confines of the epithelial cells. The location of HIV virions in the monolayer was assessed by their proximity to the plasma membrane protein occludin, stained in yellow. The percentage of virions within 0.1 μm of the cell surface contrasted against virus further from the plasma membrane (Fig. 4B) was less than 1%, indicating that over 99% of virions were within the confines of the cell. To provide further evidence that the virus was binding to the apical surface and transported within the epithelial cell, polarized monolayers were exposed to Δenvelope HIV virions. Release of these “bald” viruses to the basolateral chamber was suppressed by greater than 99.9% (Fig. 4C), confirming that viral transport in this model is via an intracellular process.

### HIV endocytosis into the intestinal epithelial cell monolayer is caveolin and lipid raft dependent.

**FIG 5:**
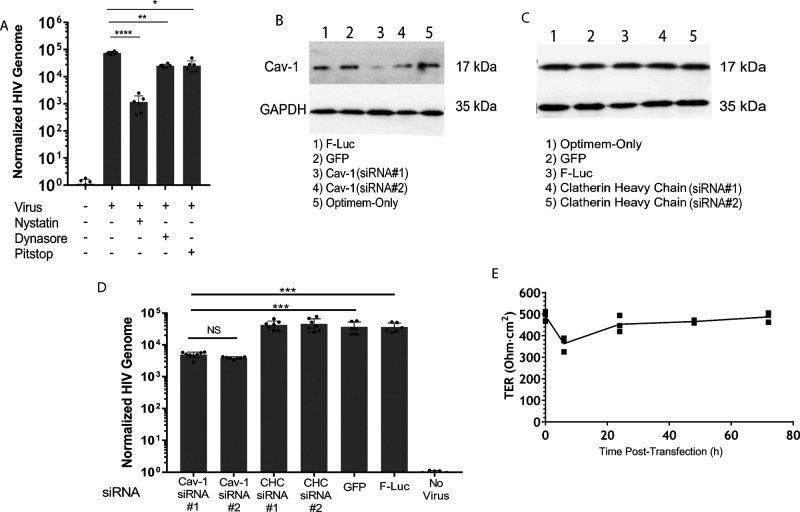
HIV uptake and release are dependent on caveolae. (A) Polarized epithelial monolayers were incubated with endocytosis inhibitors, nystatin for caveolae, Dynasore for dynamin-1, or Pitstop-2 for clathrin, added to the apical and basolateral chambers for 3 h, removed by washing with HBSS, and pulsed with GFP-HIV for 2 h. Viral RNA was isolated from the lower chamber at 24 h and quantified using RT-qPCR; *, *P* < 0.05; **, *P* < 0.01; ****, *P* < 0.0001 by ANOVA with Bonferroni correction. (B and C) Immunoblot of an epithelial cell monolayer transfected for 48 h with siRNAs directed against the targets as indicated, probed for expression of caveolin-1 and GAPDH, as an internal standard. (D) An epithelial monolayer was transfected for 72 h with two distinct siRNAs against caveolin (Cav-1) and clathrin heavy chain (CHC) and siRNAs against GFP and luciferase (F-luc), as negative controls. A 5 ng p24 equivalent of HIV was applied to the apical surface for 2 h, and viral RNA was isolated from the lower chamber at 24 h and quantified using RT-qPCR; ***, *P* < 0.001 by ANOVA with Bonferroni correction. (E) TER of transfected transwells was measured at 0, 6, 24, 48, and 72 h.

To establish a mechanism for viral uptake into the monolayer, inhibitors targeting common molecules in cellular endocytosis were studied: Dynasore (dynamin), Pitstop 2 (clathrin), and nystatin (caveolin). Polarized epithelial monolayers were pretreated in serum-free media with the endocytosis inhibitors for 3 h at the manufacturer’s recommended concentration. TER was measured after inhibitors were removed to ensure the monolayers were intact (Fig. S8). After the pretreatment, the cells were washed with HEPES-buffered saline solution (HBSS) once and then incubated with GFP-HIV for 2 h. The basolateral medium of the transwell was harvested after 24 h and the amount of viral transport was measured by RT-qPCR (Fig. 5A). Nystatin is the most effective inhibitor of HIV transport, indicating that caveolin and its related pathways are involved. Dynasore and Pitstop-2 did cause a significant reduction in HIV release (10% and 15%, respectively), suggesting that HIV is capable of utilizing other pathways of endocytosis, although their biological relevance is unknown. To confirm the role of caveolin in HIV uptake and release from the monolayer, polarized epithelial monolayers in transwells were transfected with siRNA for 48 h to silence clathrin, caveolin, GFP, and firefly-luciferase, the latter two as negative controls (Fig. 5B and C). The degree of siRNA silencing for caveolin was confirmed via immunoblot (Fig. 5B), estimated to be about 40% for Cav-1 (siRNA 1) and 45% for Cav-1 (siRNA 2). Sequences can be found in Table S1. Clathrin heavy chain siRNA transfection did not affect Cav-1 expression (Fig. 5C). To determine the effect of siRNA silencing on HIV release, transfected transwells were exposed to 5 ng of p24 of HIV in the apical chamber for 2 h, and the transwells were incubated for 24 h. Medium from the basolateral chamber was collected for viral RNA purification and RT-qPCR analysis. Results show that siRNA silencing of the caveolin-1 gene causes a significant drop in HIV release into the basolateral chamber, confirming the role for caveolin-mediated endocytosis during HIV transport (Fig. 5D). TER of transfected monolayers was recorded over the 72 h of the experiment, revealing that transfection did not disrupt the monolayer integrity (Fig. 5E).

**FIG 6:**
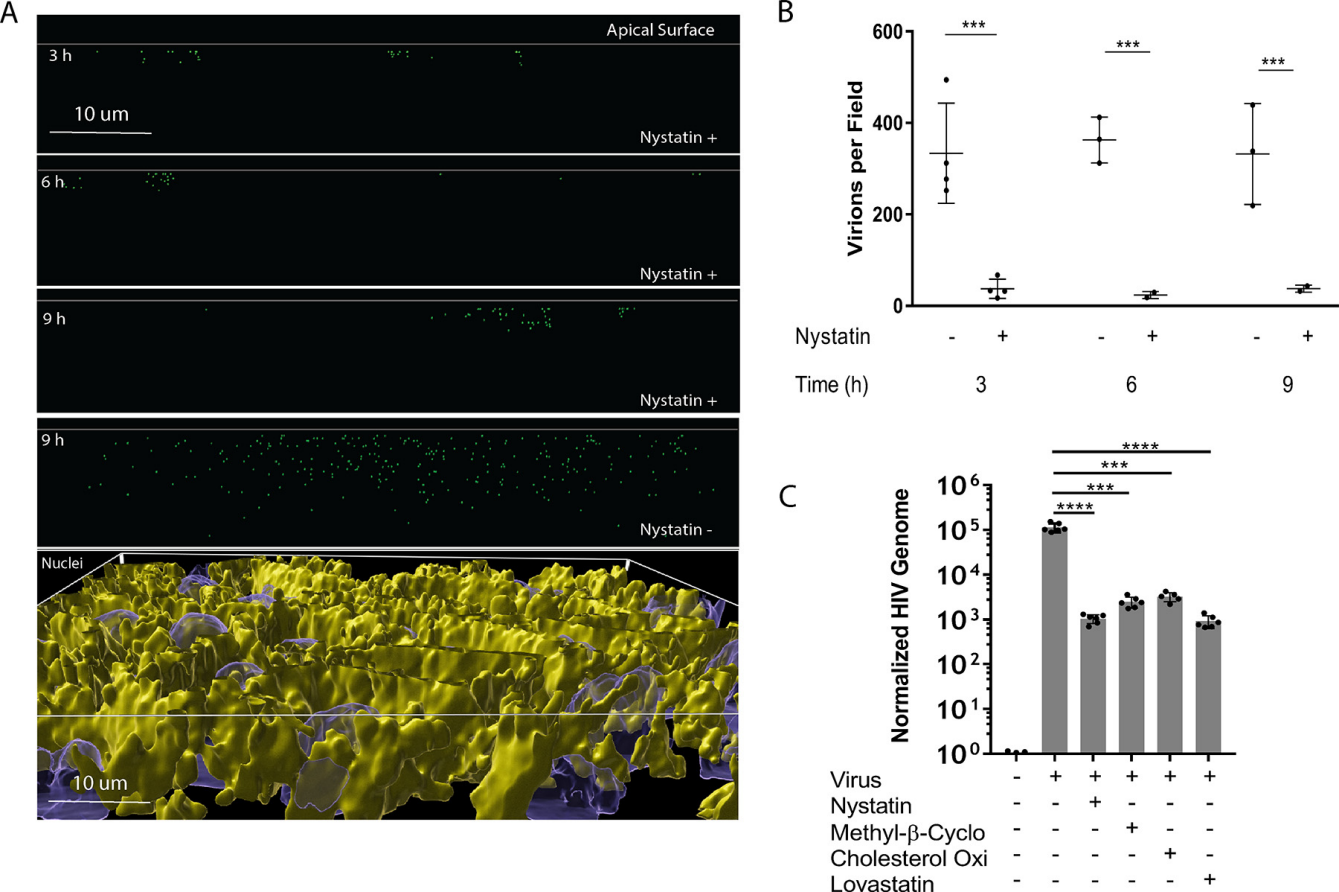
Endocytosis of HIV at the apical surface is dependent on lipid rafts. (A) Polarized epithelial monolayers were left untreated (nystatin−) or treated with nystatin for 3 h, washed, and pulsed with GFP-HIV for 2 h, and images were taken at 3, 6, and 9 h. 3D visualizations of the monolayer in the XZ plane are shown. A top-down 3D visualization in the bottom panel is stained for occludin in yellow and nuclei in blue, showing an intact, single cell depth monolayer. (B) Individual virions were counted in XZ plots; one field is 110 μm by 110 μm by 13 μm (*n* = 4; ***, *P* ≤ 0.001 by pairwise *t* test). (C) Inhibitors of lipid rafts that modulate caveolae formation (methyl-β-cyclodextrin, cholesterol oxidase, and lovastatin) were added to a polarized epithelial monolayer for 3 h, removed by washing with HBSS, and pulsed with GFP-HIV for 2 h. HIV release into the lower chamber was measured by RT-qPCR at 24 h; ***, *P* < 0.001; ****, *P* < 0.0001 by ANOVA with Bonferroni correction.

XZ plots of nystatin-treated monolayers analyzed by 3D visualization reveal that limited numbers of viruses clustered near the apical surface of the monolayer over time and less virus was present deeper in the monolayer (Fig. 6A). A top-down view of the culture confirms the presence of a single epithelial cell layer within the polarized monolayer (Fig. S9). Quantification of these XZ plots shows that the reduction of virions within the monolayer after nystatin treatment is significant (Fig. 6B). To investigate the role of lipid rafts in caveolin-mediated endocytosis of HIV, the structure of rafts was disrupted by extracting or modifying cholesterol. Methyl-β cyclodextrin, which depletes cholesterol from lipid rafts, lovastatin, an HMG-CoA reductase inhibitor, and cholesterol oxidase, which converts cholesterol to cholesterone, all inhibited HIV transport across the epithelial monolayer by at least 100-fold (Fig. 6C).

### HIV transport through the monolayer is dependent on microtubules.

**FIG 7:**
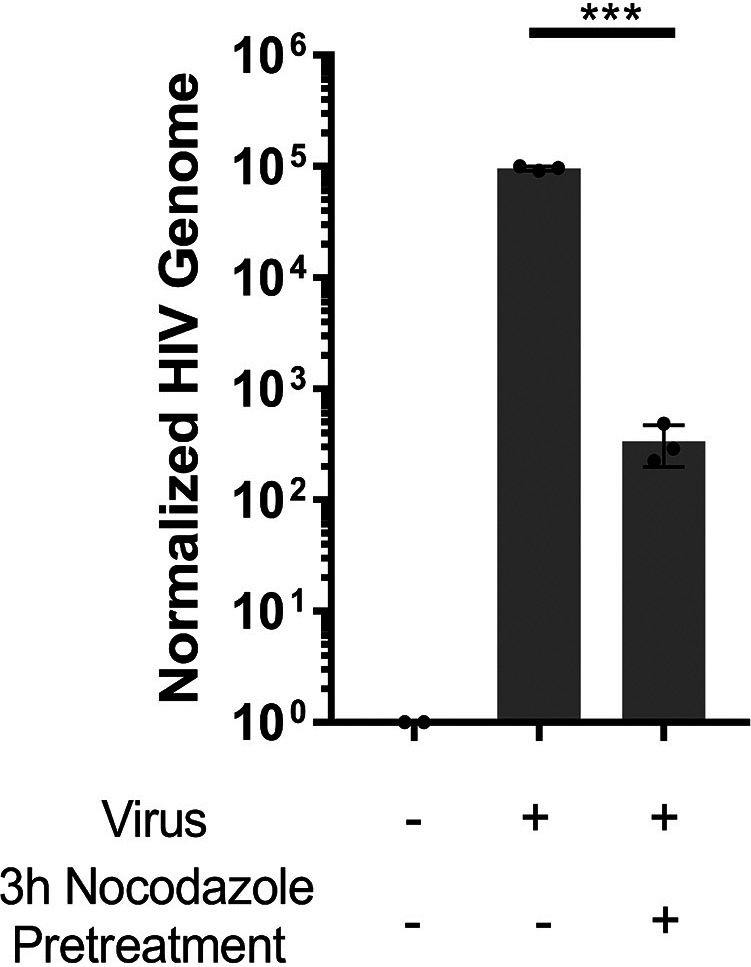
Release of HIV from the basolateral surface is dependent on tubulin polymerization. A polarized epithelial monolayer was pretreated for 3 h with nocodazole, then 5 ng p24 equivalent of GFP-HIV was added to the apical surface for 2 h, and viral RNA was isolated from the lower chamber at 24 h and quantified using RT-qPCR. ***, *P *< 0.001 by ANOVA with Bonferroni correction.

To elucidate the mechanism of transport used by HIV, the monolayer was pretreated for 3 h with nocodazole, a compound that inhibits the polymerization of microtubules. After treatment with nocodazole, 5 ng of p24 of GFP-HIV was exposed to the apical surface of the monolayer for 2 h and the concentration of the viral genome in the lower chamber was determined by RT-qPCR at 24 h. Pretreatment with nocodazole resulted in >99% decrease in viral transport and release into the basolateral chamber (Fig. 7). TER was measured after nocodazole was removed to ensure the monolayers were intact (Fig. S10).

### HIV within the monolayer is degraded over time.

**FIG 8:**
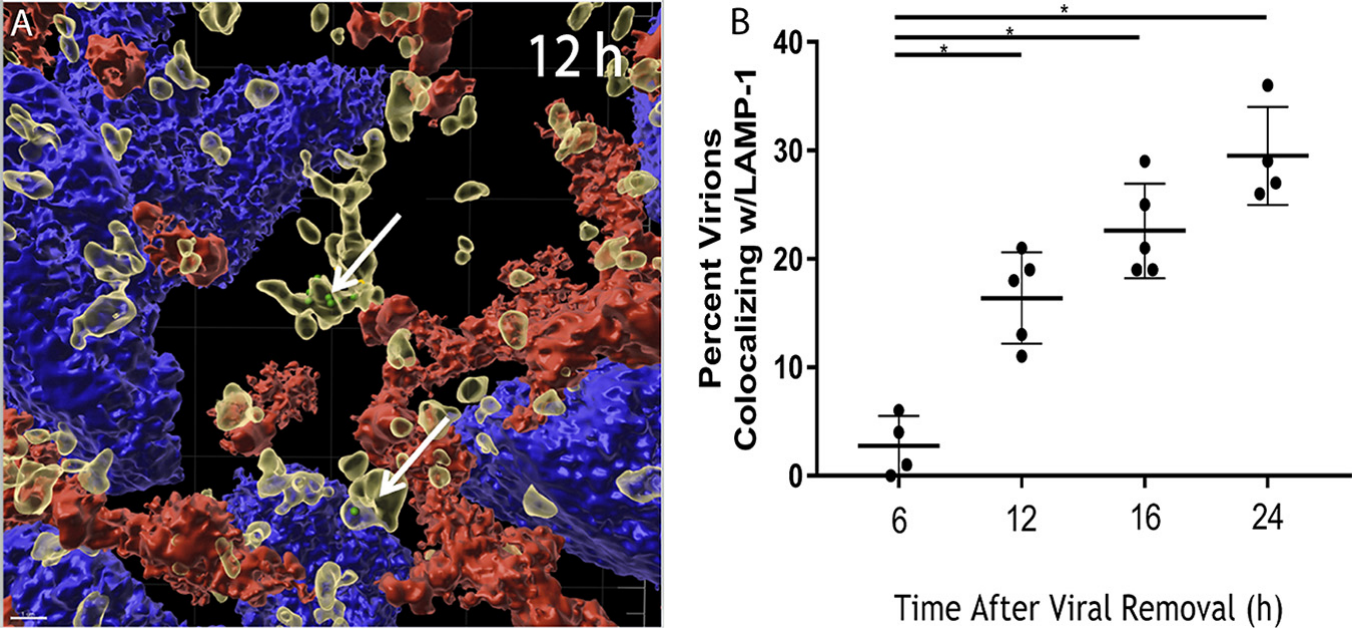
HIV within the epithelial monolayer is infectious and colocalizes with Lamp-1. (A) Polarized epithelial monolayers were treated apically with 5 ng p24 equivalent of GFP-HIV for 2 h. The virus was removed, and the monolayer was incubated for 12 h, fixed, and stained for LAMP-1 (yellow), occludin (red), and DAPI (blue). 3D visualization of a z-stack recorded at 12 h, using IMARIS. (B) Quantification of GFP-HIV and LAMP-1 colocalization within 0.1 μm at 6, 12, 16, and 24 h. Each data point represents quantitation from distinct microscopic fields from different membranes. *, *P* < 0.05 by ANOVA with Bonferroni correction.

Quantification of virions revealed that virus levels within the monolayer decrease over time (Fig. 2D), suggesting the possibility for intracellular viral degradation. To investigate the mechanism for the loss of HIV within cells of the epithelial monolayer, the apical surface of polarized monolayers was exposed to 5 ng of p24 of GFP-labeled HIV for 2 h and then allowed to incubate for 6, 12, 16, or 24 h. The monolayers were fixed and stained for occludin and lysosomal-associated membrane protein 1 (LAMP-1), a protein that resides primarily in lysosomal membranes. Staining for occludin and LAMP-1 demonstrated that the monolayers were confluent (Fig. S11A) and that HIV associated with LAMP-1+ vesicles at 6 (Fig. S11B), 12 (Fig. 8A), and 24 h (Fig. S11C), respectively. Quantification of GFP-HIV colocalizing with LAMP-1 was measured for their proximity (Fig. 8B). The findings reveal that starting at 12 h, the time at which viral concentrations within the cell decline, there is a significant increase in the number of HIV particles found within 0.1 μm of the LAMP-1+ signal, consistent with accumulation of the virus in lysosomal vesicles.

## DISCUSSION

Current thinking of HIV passage across the colonic epithelial monolayer in gay and bisexual men suggests three methods of HIV translocation: colonic tears, paracellular diffusion, and transcellular transport (34). While the relative balance among these routes is still undetermined, the mechanism for transcellular transport is largely undefined. Initial studies implicated HIV-infected lymphocytes capable of cell-to-cell viral transmission in supporting transcytosis across an intact epithelial monolayer without infection (14). As current literature indicates that seminal HIV is cell free (35), we defined the mechanism of cell-free HIV transcytosis across an intact epithelial monolayer. Our results indicate that HIV release from the basolateral surface of the monolayer increases in an exponential manner, suggesting cooperativity among virions. We propose that this cooperativity is due to virions stabilizing endocytic vesicle formation on the apical plasma membrane, resulting in enhanced endocytosis. This concept of viral-mediated endosome stabilization is further supported by our observation that monolayer transport of HIV can be saturated. When we expand the maximal viral dose beyond our standardized 5 ng of p24 to 10 and 15 ng, we see a dramatic and significant, almost complete, reduction in HIV release from the monolayer (Fig. S12). We propose that this may be caused when the endocytosis pathway, normally stabilized by viral loading, becomes overloaded with virions, causing loss of function. A similar saturation of viral transport was observed with EBV virions as they transcytosed across oral epithelial cells (12).

Another key observation when HIV transcytosis was characterized in association with mononuclear cell-to-cell transmission (14) was that viral transit across an intact epithelial monolayer was complete within 30 min. In addition, lamina propria dendritic cells are capable of “sampling” luminal HIV, enabling viral passage across the epithelium on a similar time scale (36). In contrast, in a cell-free model of HIV transport, we found that HIV transcytosis was significantly slower than noted in these cell-associated systems.

3D reconstruction of monolayers revealed that the vast majority of HIV virions were found within the cells, were not observed in the paracellular space, and did not colocalize with tight junctions, indicating that these virions are in fact transported transcellularly. In addition, Δenvelope HIV is not capable of transport across the intact epithelium, revealing that the envelope of the virion is critical for viral endocytosis and transport. Lack of Δenvelope virus release into the basolateral chamber by an intact monolayer also indicates an intracellular process. Consistent with the reported mechanisms of transcytosis for other systems (12, 37), HIV transport requires energy, as shown by significantly reduced viral endocytosis at 4°C. Curiously, HIV within the epithelial cell monolayer seemed to congregate within the upper third of the cytosol, independent of the time the monolayer was imaged. We propose that due to the nature of epithelial cell morphology, namely, that the nucleus fills the bottom third of a polarized cell, virus-bound endosomes may be structurally excluded, thereby minimizing viral transport. Virions extracted from within cells of the monolayer were infectious, indicating that HIV remains intact. Since the release of virus into the basolateral chamber is highly inefficient (14) (less than 0.1% of viral input), assessing infectious viral release was below the limits of detection for the Ghost X4/R5 cells. We, therefore, propose that 12 h after viral exposure, when the number of virions begins to decline, most HIV virions within the intestinal monolayer are sequestered in LAMP-1+ lysosomes (38). This observation matches previous studies in oral and genital epithelial cells (39–42), showing that HIV virions undergoing transcytosis across a polarized monolayer are sequestered in late endosome/lysosomes for up to 9 days postintroduction (19, 20).

During canonical HIV infection of a T cell, trimeric gp120 binds initially with the CD4 receptor on the cell surface, exposing a binding site for a coreceptor which can be either the CCR5 or CXCR4 chemokine receptors (43, 44). Formation of this complex triggers an invagination to form around the virion on the T cell surface. A clathrin-bound vesicle forms, and the virion is endocytosed, to be uncoated and complete the infection process (44). Infection does not occur in colonic epithelial cells since they do not naturally express CD4 and have low expression of CCR5 and CXCR4 (3); thus, HIV does not trigger the infection-specific endocytosis process in these cells. Previous studies have shown that galactosylceramide and heparan sulfate proteoglycans are possible adhesion molecules for HIV on the epithelial cell surface (14, 16, 45). We propose that by binding to these alternate receptors, HIV triggers the formation of a caveolae-invagination lined with cav-1 and cav-2 dimers. Both galactosylceramide and the heparan sulfate proteoglycan family of cell surface receptors are reported to be associated with cholesterol-rich lipid rafts (46–48), and successful caveolae formation is dependent on lipid rafts (48–51). Removing cholesterol from these rafts, disrupting cholesterol production, or converting cholesterol to the more rigid molecule, cholesterone, inhibits the ability of cav-1 to incorporate into the raft domains (52), resulting in improper aggregation of caveolae and reduced viral endocytosis. In contrast to clathrin-coated vesicles, caveolin-coated vesicles do not trigger the uncoating of the HIV particle, leading instead to viral transcytosis or colocalization with LAMP-1+ vesicles.

The vast majority of endocytosed virions remain within the cell (20), directed toward sequestration in vesicles and potential degradation by lysosomes. During the course of this study, we were unable to define differences between the virions that were successfully released from the basolateral membrane and those that remained within the cell. We hypothesize that virions that are successfully transported across the monolayer interact randomly with an endosome destined to be transported along the microtubules toward the basolateral surface and the remainder undergo endosomal fusion with a lysosome. Since we show that HIV enters epithelial cells by endocytosis, it can be assumed that downstream mechanisms of endosomal sorting are also used.

One limitation of this study is the use of a colonic adenocarcinoma cell line to establish the monolayer. A primary culture air-liquid interface more eloquently simulates the gut environment and can support the differentiation of multiple progenitor cell types (53). As this technology improves to support viral exposure, future studies are needed to confirm and expand our observations. In summary, polarized colonic epithelial cells facilitate HIV transcytosis using caveolin-based, lipid raft-dependent endocytosis. HIV transcytosis along microtubules and release of intact virions into the lamina propria can lead to productive infection. Thus, selectively blocking this transport pathway and identifying the surface receptor used by HIV to trigger transcytosis are viable pharmaceutical targets to prevent the spread of HIV.

## MATERIALS AND METHODS

### Cell culture and virus.

The colonic epithelial adenocarcinoma cell line, Caco-2, was purchased from ATCC (Manassas, VA), and polarized monolayers were grown in complete media consisting of EMEM (Corning, Tewksbury, MA) with 10% fetal bovine serum (FBS, Corning) on transwell inserts (Corning; 0.4-μm pore size, 12-well inserts). The barrier integrity of the monolayer was determined by transepithelial resistance (TER), measured with the EVOM and EVOM2 voltohmeters (World Precision Instruments, Sarasota County, FL). Cultures that did not meet the benchmark TER of 200 Ω·cm^2^ or higher were not used.

GFP-HIV, a generous gift of John Tilton (54), was assembled from a two-plasmid system (22) via transfection of HEK-293T cells (ATCC) using calcium phosphate (54). The conditioned medium was harvested 72 h and 96 h posttransfection, and cell debris was removed by centrifugation at 1,200 rpm for 5 min and filtration through a 0.45-μm filter (Genesee Scientific, San Diego, Ca). Virus was harvested by ultracentrifugation for 2 h at 25,000 × *g* in an SW 32 Ti Rotor (Beckman Coulter Life Sciences, Indianapolis, IN). Virus was also purified and concentrated by tangential flow filtration (TFF) using KrosFlo KR2i TFF System (Repligen, Waltham, MA) fitted with a MicroKros 20 cm 500KD 0.5 mm column (Repligen). ΔEnvelope HIV was created using the same method as above, removing the plasmid that carries the ENV gene.

### Translocation of virus through the monolayer and treatment with pharmacological agents.

Polarized Caco-2 monolayers plated in transwells were incubated with graded concentrations of HIV for 2 h in 20 μL of Eagle’s medium essential medium (EMEM) on their apical membrane. The virus in the apical chamber was removed by aspiration and replaced with 100 μL of fresh media plus 10% fetal bovine serum (FBS). To inhibit endocytosis or disrupt lipid rafts, polarized Caco-2 cells were incubated with the following inhibitors at the manufacturer’s recommended concentrations: 20 μM Pitstop 2 (Abcam, Branford, CT), 40 μM Dynasore, 12.5 μg/mL nystatin, 10 mM methyl-β-cyclodextrin, 5 μg/mL lovastatin, and 5 μg/mL cholesterol oxidase (all from Sigma-Aldrich, St. Louis, MO). Inhibitors were added to the apical and basolateral chambers of the transwell for 3 h in serum-free media. The cells were washed with HBSS (Lonza, Rockville, MD) and incubated with GFP-HIV (5 ng of p24 equivalent virus) for 2 h, as noted above. After removal of the virus, the monolayers were incubated for 2 to 24 h.

### Deconvolution immunofluorescent microscopy and 3D visualizations.

The polarized Caco-2 monolayer was washed with HBSS thrice and fixed using 4% paraformaldehyde for 15 min. The transwell membranes were excised from their plastic fitting with a razor, and the cells were permeabilized with 0.1% saponin in phosphate-buffered saline (PBS) for 30 min. Occludin was detected using mouse anti-occludin antibody (Invitrogen, Carlsbad, CA) and Cy3 AffiniPure goat anti-mouse IgG (H+L) (Jackson ImmunoResearch, West Grove, PA). Actin was bound by Alexa Fluor 680 phalloidin (Thermo Fisher Scientific, Waltham, MA), and the nuclei were stained with 4′,6-diamidino-2-phenylindole (DAPI, Thermo Fisher). Cells were imaged by deconvolution microscopy in a Delta Vision microscope (General Electric, Boston, MA). 3D visualization was performed using IMARIS software (Bitplane, Zurich, Switzerland). Viral quantification and colocalization were assessed using on-board IMARIS utilities.

### RT-qPCR.

Quantitation of the viral genome was determined by RT-qPCR. A total of 5 μL of purified Caco-2 cellular RNA at concentration 500 ng/μL was spiked into the medium in the lower chamber of the transwells. Medium from the lower chamber was harvested, and RNA was isolated with a QIAamp viral RNA minikit (Qiagen, Frederick, MD). Reverse transcription of the viral and host RNA was performed using the high-capacity RNA-to-cDNA kit (Applied Biosystems, Foster City, CA). qPCR for the reverse transcriptase gene of HIV and the GAPDH gene of the Caco-2 cells was performed using SYBR green (Applied Biosystems) in a Quant Studio 3 thermocycler (Applied Biosystems). HIV-RT gene forward: GTGCCCGTCTGTTGTGTGAC. HIV-RT gene reverse: GGCGCCACTGCTAGAGATTT. GAPDH gene forward: AAATTGAGCCCGCAGCCT. GAPDH gene reverse: TAAAAGCAGCCCTGGTGACC.

### siRNA transfection.

siRNA transfection was performed using Lipofectamine RNAiMAX transfection reagent kit (Thermo Fisher) on polarized Caco-2 cells plated in transwells. TER was measured before and after each step. Custom and premade siRNA (Millipore Sigma, Cat no. NM_001753) targeting caveolin, clathrin, f-luciferase, and GFP genes were transfected into the cells and allowed to incubate for 48 h. A sample of untreated and treated cells was taken for immunoblot analysis. At 48 h, virus was introduced to the apical chamber of the transwell for 2 h and then removed. Viral release into the lower chamber was measured as described previously. siRNA sequences are found in Table S1.

### Immunoblots.

Caco-2 cells plated in transwells that were previously transfected with siRNA were lysed using 10 μL radioimmunoprecipitation assay (RIPA) buffer (Thermo Fisher) and 0.5 μL DNase-1 solution (Thermo Fisher). The lysate was then mixed with an equal volume of 2× Laemmli sample buffer (Bio-Rad, Hercules, CA), heated at 95°C for 5 min, and fractionated by electrophoresis on a 10% polyacrylamide gel at 50 V for 1 h and then 120 V until dye front was 6 mm from the bottom of the glass plates encasing the gel. A polyvinylidene difluoride (PVDF; Bio-Rad) membrane was activated in methanol for 1 min, and protein was transferred from the gel to the membrane overnight at 30 V at 4°C. The membrane was blocked with 5% milk in Tris-buffered saline with 0.1% Tween (TBST; Millipore Sigma) and probed with anti-caveolin-1 antibody (Abcam, 1:1,000 dilution) and anti-GAPDH antibody (Thermo Fisher 1:10,000 dilution) in 5% milk/TBST. Membranes were washed three times in TBST for 15 min. Secondary antibodies targeting mouse (Jackson ImmunoResearch, Cat. no. 115-165-062, 1:10,000 dilution) and rabbit (Abcam, Cat. no. ab97075, 1:10,000 dilution) in 2.5% milk/TBST were incubated with the membrane for 2 h at room temperature. Membrane was washed three more times in TBST for 15 min each. A 1:1 mixture of peroxide chemiluminescent detection reagent and luminol/enhancer solution (Advasta, San Jose, CA) was added to the membranes for 10 min, and then the membranes were imaged using ImageQuant 4000 (GE Healthcare, Chicago, IL).

### HIV infectivity.

Polarized Caco-2 cells plated in transwells were incubated apically with 5 ng of p24 of HIV for 2 h, and then virus was removed as described previously. After 12 h, cells were lysed by scraping and sonication. The lysate was passed through an 0.4-μm filter (ThermoFisher) and centrifuged at 1,500 rpm for 5 min. Ghost (3) X4/R5 cells (NIBSC, South Mimms, Hertfordshire) were cultured for 5 days in 12-well plates until reaching ∼60% confluence, incubated with the monolayer lysate for 72 h, and imaged for GFP expression using deconvolution microscopy.

### Statistical analyses.

Statistical evaluation was performed via analysis of variance (ANOVA) for multiple comparisons using a Bonferroni correction or the Student’s *t* test using Prism 8.0 (GraphPad Software, San Diego, CA), as indicated in the figure legends. All values are provided as mean ± standard deviation (SD). *P* values less than 0.05 were considered significant. Each dot represents the average of at least 3 replicates.
