# Supplementary material for: Impact of Caveolin-Mediated Endocytosis on the Trafficking of HIV within the Colonic Barrier
Source: J Virol. 2022 Mar 17;96(7):e00202-22. doi: 10.1128/jvi.00202-22 (PMC9006927; doi:10.1128/jvi.00202-22)
Supplement: Supplemental file 1 — Fig. S1 to S2; Supplemental Movie Legend; Table S1. Download jvi.00202-22-s0001.pdf, PDF file, 1.0 MB [file jvi.00202-22-s0001.pdf]

Trafficking or degradation of HIV within the Colonic Barrier is dependent on Caveolin-mediated endocytosis

Running Title: HIV transcytosis is caveolin and tubulin dependent

Alex Anwar[1], Michelle Helou[1], Jessica Hervol[1] and Alan D. Levine[1,2]

Departments of [1]Molecular Biology and Microbiology, [2]Pathology, [2]Pharmacology, [2]Medicine, [2]Pediatrics, [2]Biological Sciences, and [2]CWRU Center for Excellence on the Impact of Substance Use on HIV, Case Western Reserve University, Cleveland, OH, 44106, USA

Supplemental Figure Legends

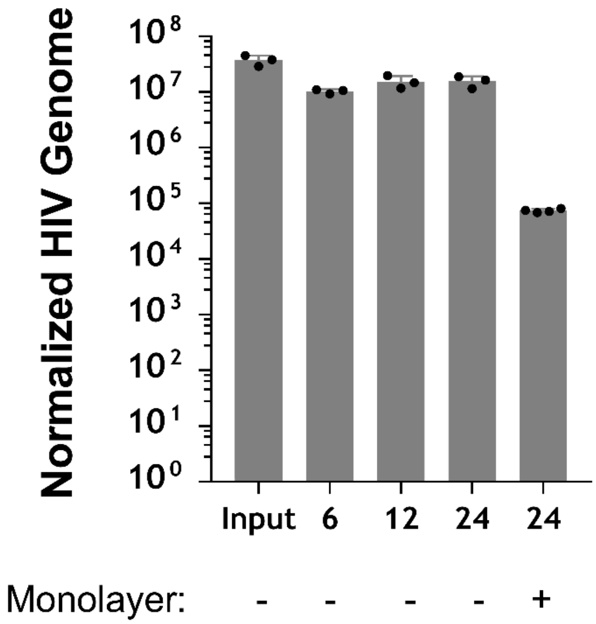

Supplemental Figure 1: HIV diffuses freely across the transwell micro-porous membrane in the absence of an epithelial monolayer. 5 ng p24 of GFP-HIV was added to the upper chamber of a transwell culture dish for 2 h in the absence and presence of an epithelial cell monolayer. Virus is then removed by washing and fresh media is added. The culture was incubated for 24 h, and viral concentrations in the lower chamber were measured by RT-qPCR.

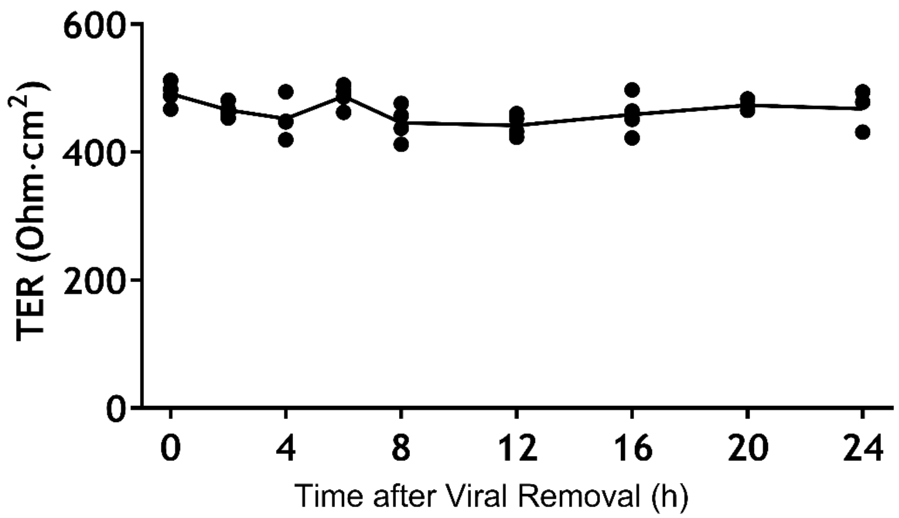

Supplemental Figure 2: TER of a polarized epithelial monolayer remains unchanged over the 24 h period when incubated with GFP-HIV.

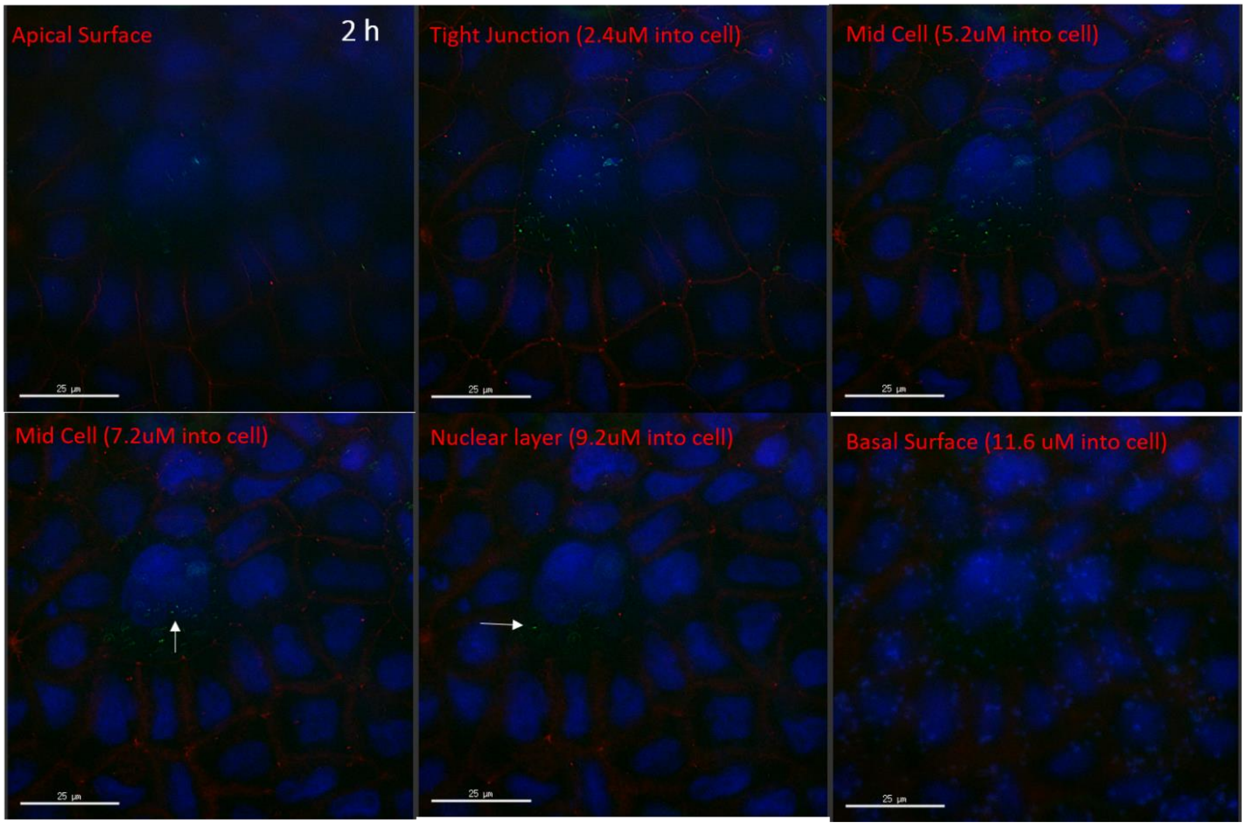

Supplemental Figure 3: **GFP-HIV particles are found throughout the epithelial cell monolayer at 2 h**. Viral particles (white arrows) were imaged using deconvolution fluorescent microscopy (Green = GFP-HIV, Red = Occludin, Blue = DAPI).

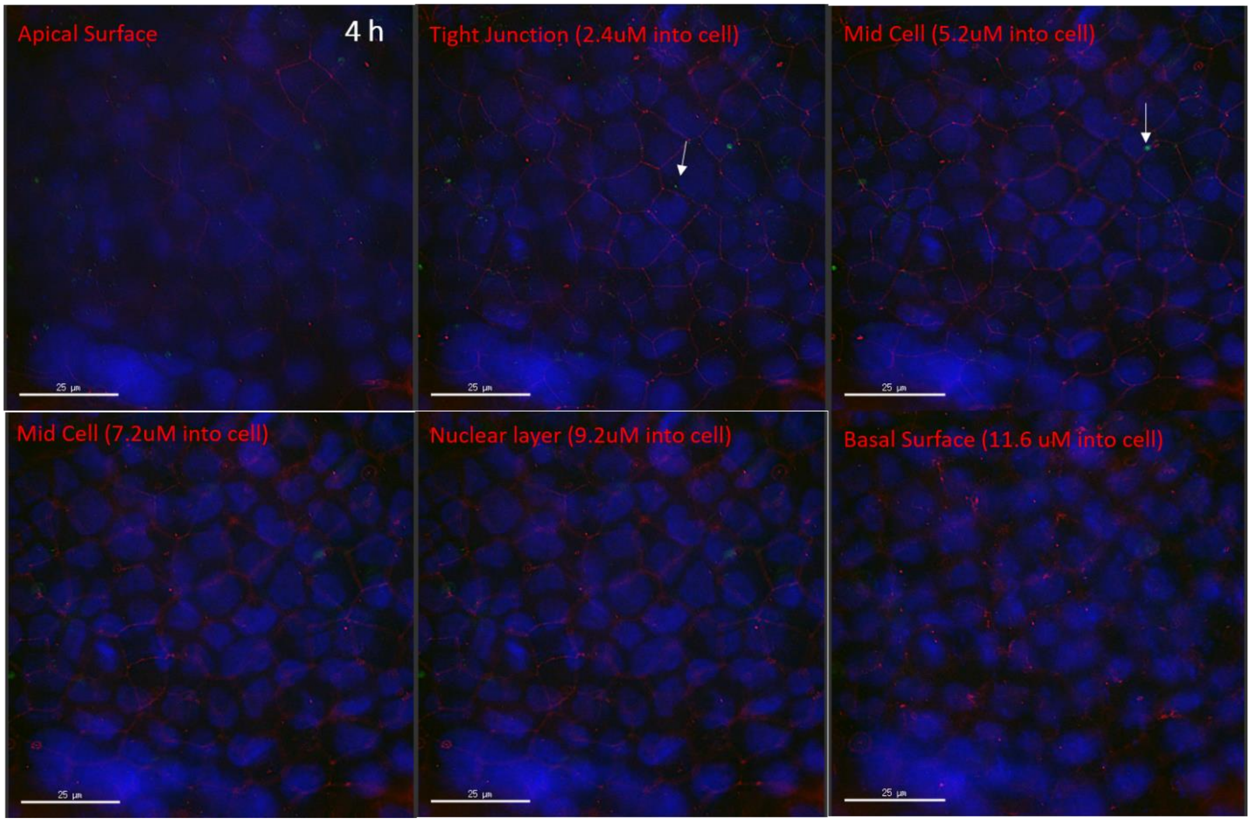

Supplemental Figure 4: **GFP-HIV particles are found throughout the epithelial cell monolayer at 4 h.** Viral particles (white arrows) were imaged using deconvolution fluorescent microscopy (Green = GFP-HIV, Red = Occludin, Blue = DAPI).

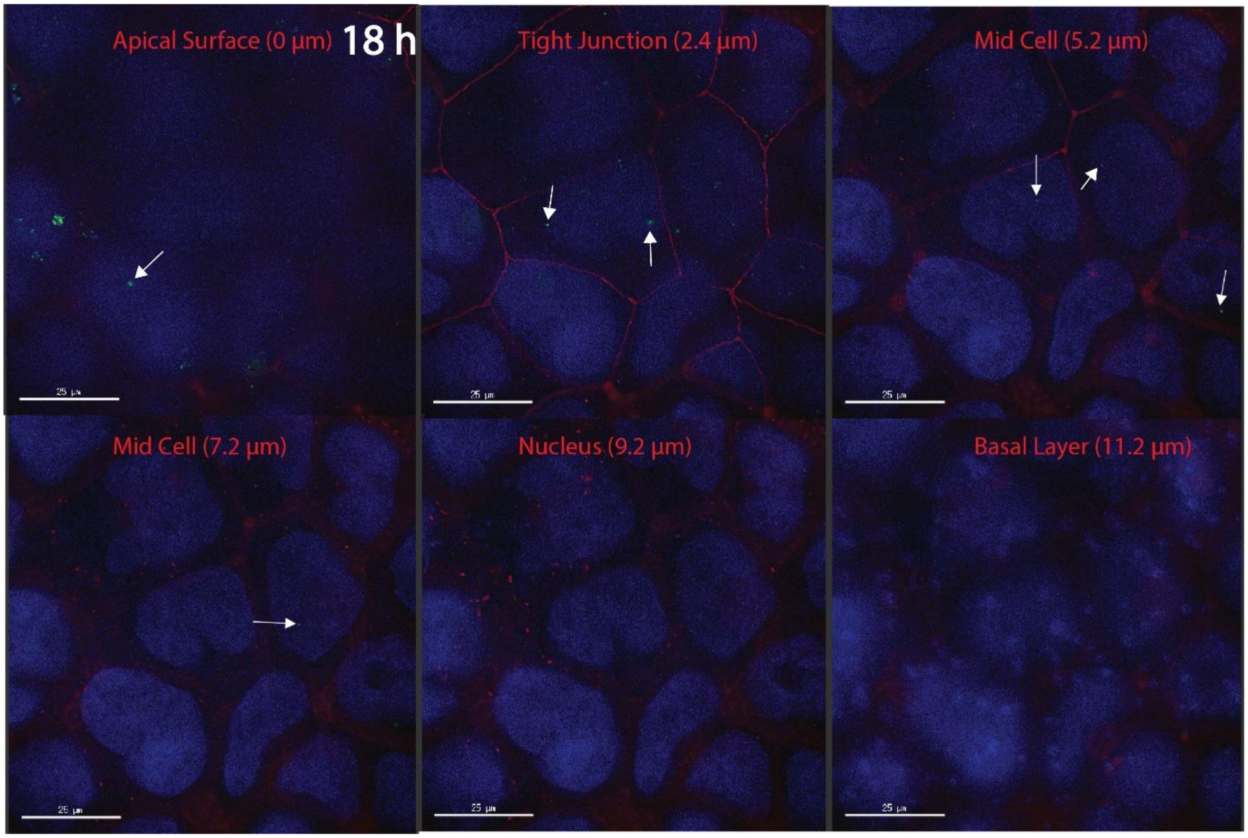

Supplemental Figure 5: **GFP-HIV particles are found throughout the epithelial cell monolayer at 18 h**. Viral particles (white arrows) were imaged using deconvolution fluorescent microscopy (Green = GFP-HIV, Red = Occludin, Blue = DAPI).

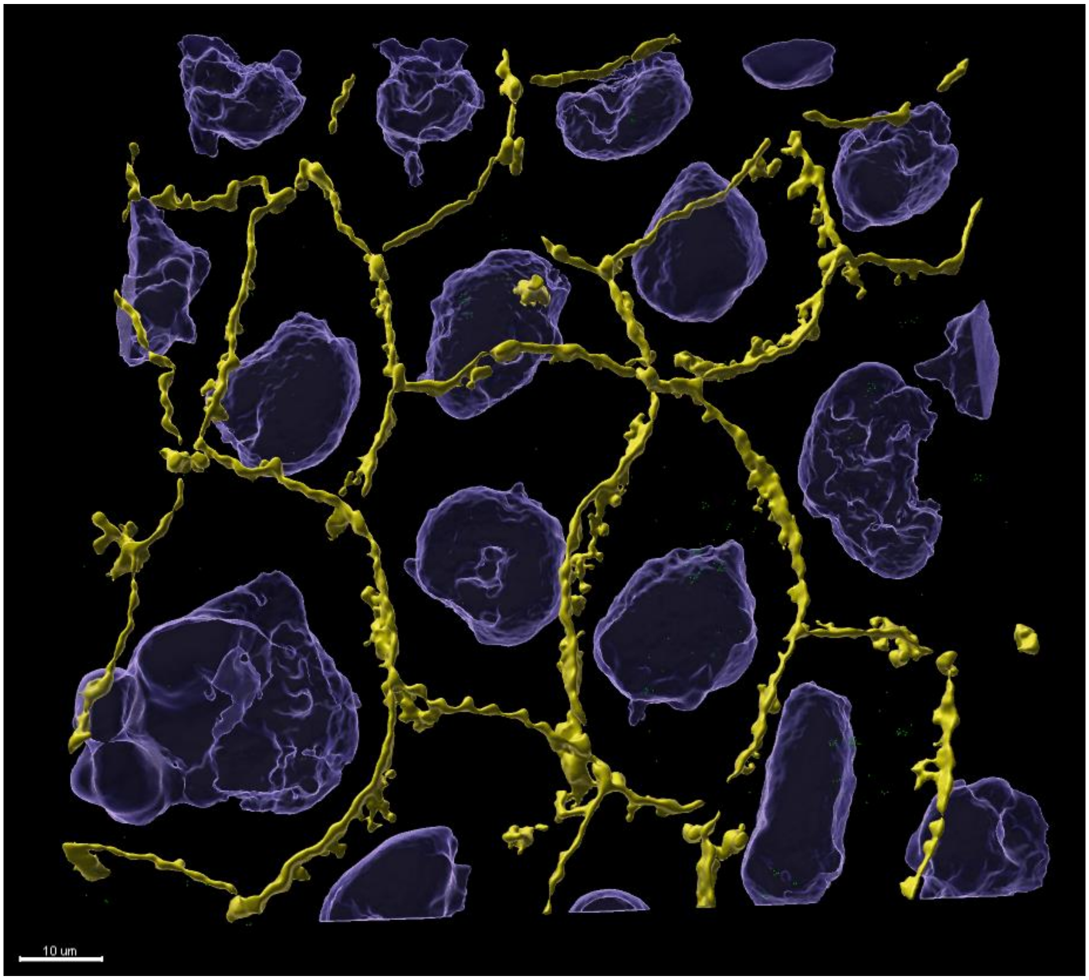

Supplemental Fig 6: A single epithelial cell layer within the polarized monolayer. A top-down view highlighting the presence of a single epithelial cell layer within the polarized monolayer, representative of monolayers used for deconvolution microscopy (Yellow = Occludin, Blue = DAPI).

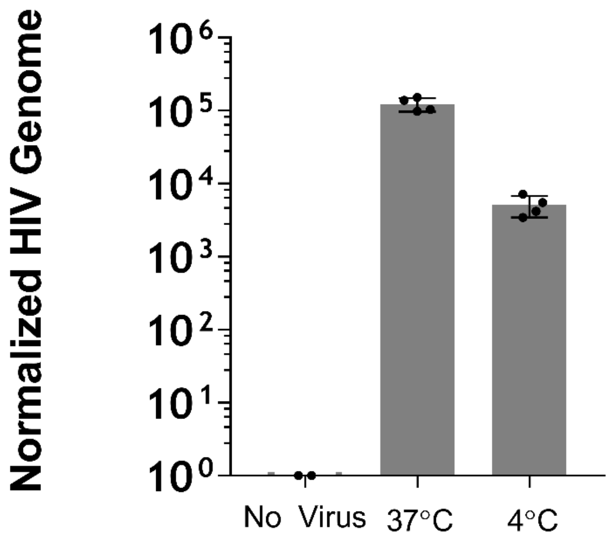

Supplemental Figure 7: HIV transport across a polarized epithelial monolayer is temperature dependent. Polarized epithelial monolayers were incubated with 5 ng p24 of GFP-HIV for 2 h, during which the cells were either placed at 4°C or 37°C. Virus is then removed by washing and fresh media is added. The cells incubated for 24 h, and viral concentrations in the lower chamber were measured by RT-qPCR.

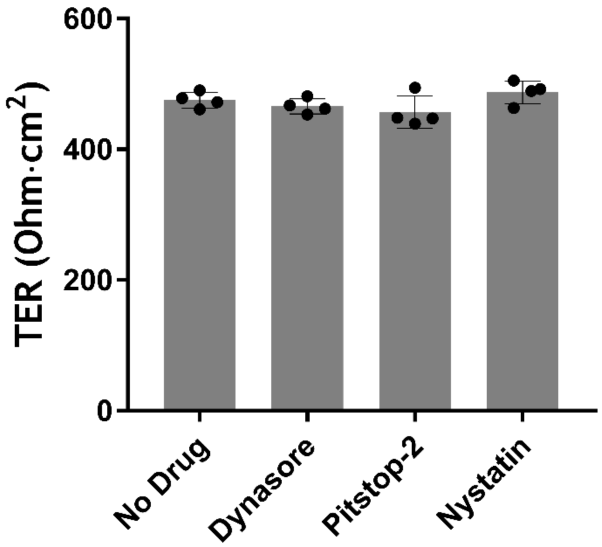

Supplemental Figure 8: Endosomal inhibitors do not affect monolayer integrity. TER of a polarized epithelial monolayer remains unchanged after incubation with the endosomal inhibitors, dynasore, pitstop-2, and nystatin, measured at 3 h.

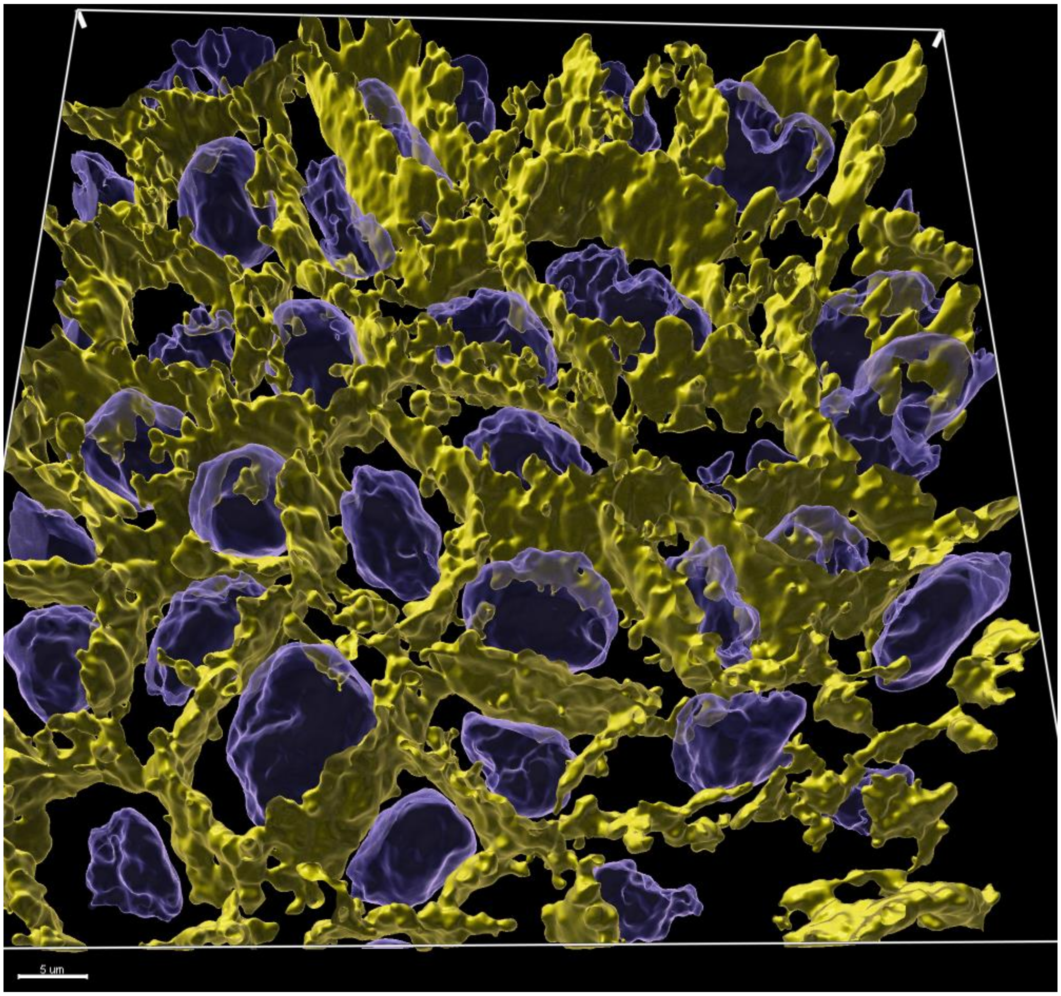

Supplemental Figure 9: **A single epithelial cell layer within the polarized monolayer.**

A top-down view highlighting the presence of a single epithelial cell layer within the polarized monolayer (Yellow = Occludin, Blue = DAPI).

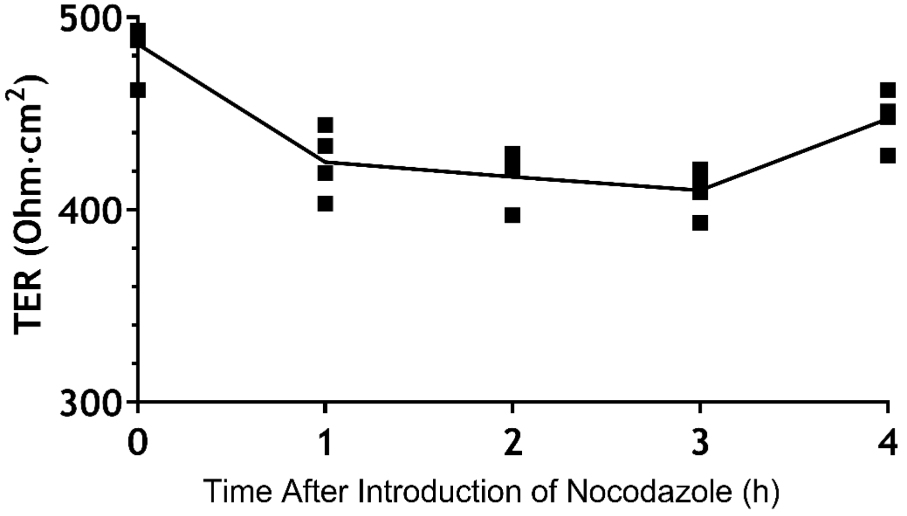

Supplemental Figure 10: Nocodazole does not affect monolayer integrity. TER of a polarized epithelial monolayer remains unchanged after incubation with Nocodazole.

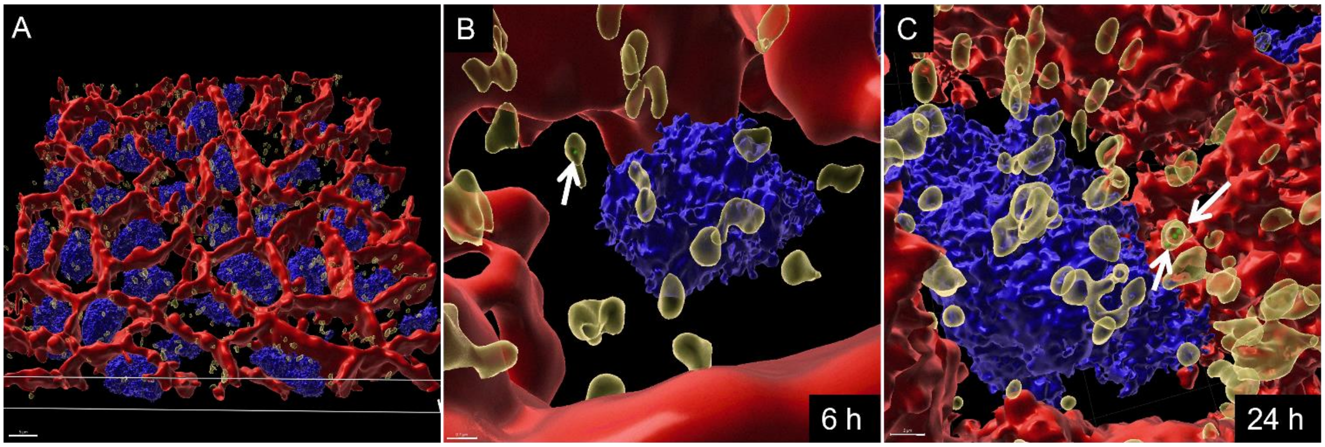

Supplemental Figure 11: Monolayers used for LAMP-1 co-localization were intact, and GFP-HIV co-localization with LAMP-1 increases over time. A top-down view of an intact polarized epithelial monolayer used in LAMP-1 staining, highlighting the presence of a single epithelial cell layer (**A**). Co-localization of GFP-HIV particles with LAMP-1+ endosomes after 6 and 24 h incubation (**B**, **C** respectively). (Green = GFP-HIV, Red = Occludin, Yellow = LAMP-1, Blue = DAPI)

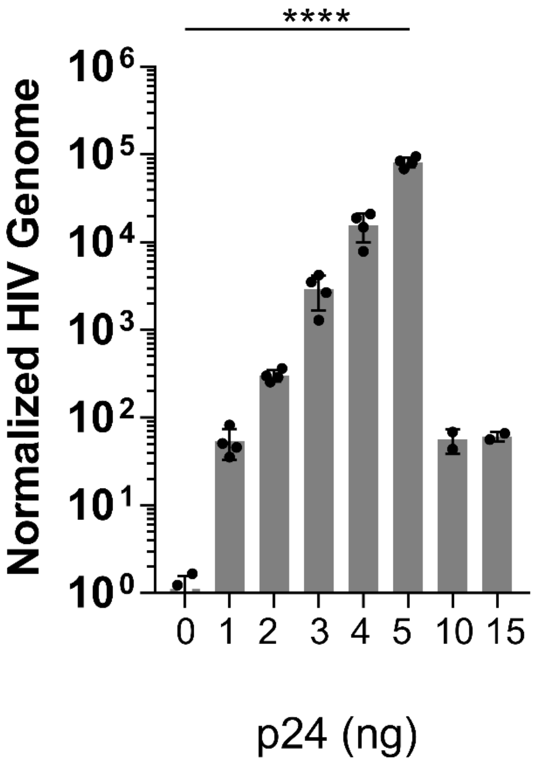

Supplemental Figure 12: Oversaturation of a monolayer with GFP-HIV causes loss in transport. HIV release into the basolateral chamber is dramatically reduced when viral input exceeds 10 ng of p24 of HIV.

Supplemental Movie Legend

Supplemental movie 1: GFP-HIV transport through the monolayer at 6 h. Movie recorded from the apical surface to the basolateral surface in 0.4 µm slices. (Green = GFP-HIV, Red = Occludin, Blue = DAPI).

Supplemental Table 1

**siRNA Target**	**Sequence**
Caveolin-1 (Cav-1 siRNA#1)	5'-AACCAGAAGGGACACACAG-3'
Caveolin-1 (Cav-1 siRNA#2)	Millipore Sigma, Cat# NM_001753; SASI_Hs01_00199504
Clatherin Heavy Chain (CHC siRNA#1)	5'-AAGCUGGGAAAACUCUUCAGA-3'
Clatherin Heavy Chain (CHC siRNA#2)	5′-AACAUUGGCUUCAGUACCCUG-3′
GFP	5′-GUUCAGCGUGUCCGGCGATT-3′
Firefly-Luciferase	5'-CUUACGCUGAGUACUUCGATT-3'
